# Editorial: Progress in Pathogen Identification Based on Mass Spectrometry

**DOI:** 10.3389/fcimb.2021.813133

**Published:** 2022-01-14

**Authors:** Junping Peng, Yi-Wei Tang, Di Xiao

**Affiliations:** ^1^ National Health Commission (NHC) Key Laboratory of Systems Biology of Pathogens, Institute of Pathogen Biology, Chinese Academy of Medical Sciences & Peking Union Medical College, Beijing, China; ^2^ Key Laboratory of Respiratory Disease Pathogenomics, Chinese Academy of Medical Sciences and Peking Union Medical College, Beijing, China; ^3^ Department of Medical Affairs, Danaher Diagnostic Platform/Cepheid (China), New York, NY, United States; ^4^ State Key Laboratory of Infectious Disease Prevention and Control, Collaborative Innovation Center for Diagnosis and Treatment of Infectious Diseases, National Institute for Communicable Disease Control and Prevention, Chinese Center for Disease Control and Prevention, Beijing, China

**Keywords:** mass spectrometry, microbial identification, antimicrobial susceptibility testing, species differentiation, database

The rapid identification of microbial pathogens is essential for the diagnosis and treatment of infectious diseases and the development of targeted prevention and treatment measures. The ubiquitous spread of novel infectious agents and multi-drug-resistant bacteria has generated a pressing need to develop rapid and reliable methods for microbial identification and antimicrobial susceptibility testing, which have previously relied on traditional culture-based methods that are time-consuming and labor-intensive. The potential utility of mass spectrometry (MS)-based techniques, including matrix-assisted laser desorption/ionization time-of-flight (MALDI-TOF) MS and liquid chromatography-tandem MS (LC-MS/MS), has been widely explored (Patel, 2015; Tran et al., 2015; Sandalakis et al., 2017; Oviano et al., 2018; Li et al., 2020). In the Research Topic of “Progress in Pathogen Identification Based on Mass Spectrometry”, we covered the latest progresses of MS technologies in the field of pathogen biology. The Research Topic contains sixteen articles: thirteen reports of original research, two reviews, and one brief research report, covering various research directions from pathogen identification and procedure optimization, the analysis of antimicrobial resistance, to database extension.

Ten articles address the Research Topic of targeting proteins, lipids, or nucleic acids with MS-based methods to obtain stable, high abundance expression profiles. The profiles of the targeted bacteria or fungi are then compared with existing profiles in a database to identify strains at the genus or species level. Proper specimen preprocessing is the first key step in this procedure. Cuénod et al. optimized the factors associated with the spectral quality of MALDI-TOF MS in species identification, comparing the significant differences obtained by varying the sample preparation protocol, bacterial culture time, and the interval from calibration to detection. More specifically, to reduce the problems caused by the insufficient coverage of commercial databases and the deficiencies in the multi-step protein extraction procedure used in the routine identification of clinical filamentous fungi, Ning et al. developed two rapid protein extraction methods using focused ultrasonication and zirconia-silica beads and built an in-house spectral library for the identification of filamentous fungi. That study reported the first use of zirconia-silica beads as a sample-processing method for building an in-house library of MALDI-TOF MS data. Another optimized protein extraction method was evaluated by Dai et al. These researchers combined density centrifugation and extra chemical lysis extraction to develop a rapid and simplified protocol for the direct identification of microorganisms in positive blood cultures. Like bacterial infections, fungal infections pose a major health burden, inducing superficial mycosis or organ disease, according to the invasion site. Baumbach et al. evaluated the reliability of MALDI-TOF MS for the identification of closely related zoophilic dermatophytes, and generated a score-oriented distance dendrogram. They compared the spectra obtained under two different growth conditions, i.e., liquid broth vs solid agar medium, which indicated that the use of liquid media for species identification or master spectra generation was not superior to the use of solid medium covered with filter paper. To establish an accurate and rapid identification method for *Candida auris* infection at the species level, De Carolis et al. developed a fast and reproducible MALDI-TOF MS assay and used the Bruker Daltonics Biotyper^®^ database with *C. auris* spectrum profiles to generate a score-oriented dendrogram with a hierarchical cluster analysis. This allowed the classification of *Candida* isolates and non-*Candida* isolates according to species. In parallel research, they investigated the capacity of MS antifungal susceptibility testing (MS-AFST) to detect the susceptibility or resistance phenotypes of *C. auris* isolates, which is described in the next section. Some comparative studies have been based on different MALDI-TOF MS platforms or other proteomic analyses. Yi et al. evaluated the performance of Autof MS 1000 and Vitek MS in identifying closely related yeasts, including fourteen different species in five species complexes. The two commercial MALDI-TOF MS platforms differed in their identification capability: Autof MS 1000 showed a greater capacity for yeast identification, whereas Vitek MS was less accurate, mainly because the reference database of phylogenetically closely related yeast species was poor. In another comparative study, Kondori et al. used bottom-up proteomic approaches, LC-MS/MS, and species-specific peptide identification (shotgun proteotyping) to identify bacteria and fungi directly in a model system. The sensitivity and accuracy of the system in analyzing spiked negative blood samples and positive blood samples without further culture were compared with those of MALDI-TOF MS. This comparative study demonstrated that proteotyping-based methods, such as LC-MS, provided promising ways to detect infectious pathogens. This was confirmed in parallel by Bajaj et al. The structural diversity and species specificity of lipids and nucleic acids offer information that complements conventional protein-based MS approaches. Solntceva et al. reviewed the recent applications of MS-based lipidomics to the identification of microorganisms and the detection of antibiotic resistance. The latter is described below in detail. This review examines the future directions of MS in microbial lipidomics. Sun et al. combined MALDI-TOF MS with quantitative real-time PCR to determine the etiology of community-acquired pneumonia in the enrolled children and to identify the appropriate antibiotic therapy. That research is valuable for its provision of a comprehensive database of pathogens, including interrelated bacteria and viruses, and is extremely important in guiding antibiotic therapies based on etiology.

Antimicrobial resistance has evolved into a serious problem for public health, and rapid and accurate pathogen detection is essential for formulating effective programs of antibiotic treatment. MS-based techniques have opened another door in the field. Five articles are related in this Research Topic. Florio et al. provided a updated overview of the various methods based on MALDI-TOF MS that have been proposed, and provided well-referenced information on the antimicrobial resistance of clinically relevant bacteria. This included a method to assess β-lactamase activity by visualizing the hydrolysis of the β-lactam ring or the detection of biomarkers that correlate with drug-resistance. Wang et al. described proteins that were differentially expressed in drug-resistant and drug-susceptible *Acinetobacter baumannii* isolates, using label-free tandem mass tag labeling and a glycoproteomic analysis, to fully clarify the mechanism of antibiotic resistance. Similarly, Lu et al. investigated the failure of polymyxin B to affect *A. baumannii* by analyzing the whole membrane proteome of polymyxin-B-induced *A. baumannii* (ATCC 19606) with high resolution MS, using label-free quantitative and targeted proteome analyses to identify differentially expressed membrane proteins with nano-LC-MS/MS. In so doing, they developed a relatively rapid membrane protein extraction and preparation method. As mentioned above, Carolis et al. used a composite correlation index (CCI)-based proteomic approach to detect antifungal resistance in *Candida* species, developing a cost-effective and time-efficient method superior to conventional growth-based antifungal susceptibility testing. Similarly, Solntceva et al. confirmed the correlations between drug resistance and changes in membrane composition or the relative abundances of lipids. These studies demonstrated that several MS approaches, especially MALDI-TOF MS, provided auxiliary tools with excellent timelines and accuracy, which should allow clinicians to promptly select effective antimicrobial therapies for pathogen-based diseases.

Other researches based on the MS technology, such as database expansion and mechanism analyses, are also included. Bernhard et al. optimized sample pre-processing procedures, on the basis of which they created a set of publicly available references for the *Cryptococcus neoformans*/*gattii* species complexes for use with the MALDI Biotyper system. To examine the influence of growth media on toxin production or activity, Hille et al. used MALDI-TOF MS-based biomarker detection models to distinguish the presence or absence of secreted exotoxins in *Moraxella bovoculi* during incubation on different growth media, with or without calcium ions. Other mechanism-directed research conducted by Thorsing et al. investigated the development of effective vaccines against enterotoxigenic *Escherichia coli* (ETEC). The researchers used the MS-based method BEMAP (β-elimination of O-linked carbohydrate modifications followed by the Michael addition of 2-aminoethyl phosphonic acid) and observed an important correlation between O-linked glycosylation and the relative immunogenicity of bacterial proteins. This finding constituted a proof of concept in considering ETEC proteins for inclusion in future broad-coverage subunit vaccine candidates.

In summary, these studies included in the Research Topic quantified the advantages and potential utility of MS in bacterial identification, the analysis of antimicrobial resistance, and database expansion, and provided a scientific basis for the timely formulation of therapy options and the further improvement of patient prognoses. Because MS is a continuously developing platform, attention should be paid to strengthen the comparative analysis of different systems, with multicenter verification, to expand and refine the clinical applications of MS-based methods in diagnostic microbiology.

## Author Contributions

JP was a guest associate editor of the Research Topic and wrote the paper text. Y-WT and DX were guest associate editors of the Research Topic and edited the text, respectively. All authors contributed to the article and approved the submitted version.

## Funding

This research project is funded by the grants from the CAMS Innovation Fund for Medical Science (CIFMS, 2021-I2M-1-038).

## Conflict of Interest

The authors declare that the research was conducted in the absence of any commercial or financial relationships that could be construed as a potential conflict of interest.

## Publisher’s Note

All claims expressed in this article are solely those of the authors and do not necessarily represent those of their affiliated organizations, or those of the publisher, the editors and the reviewers. Any product that may be evaluated in this article, or claim that may be made by its manufacturer, is not guaranteed or endorsed by the publisher.
